# Ultrasensitive Determination of Malathion Using Acetylcholinesterase Immobilized on Chitosan-Functionalized Magnetic Iron Nanoparticles

**DOI:** 10.3390/bios8010016

**Published:** 2018-02-13

**Authors:** Núbia Fernanda Marinho Rodrigues, Sakae Yotsumoto Neto, Rita de Cássia Silva Luz, Flávio Santos Damos, Hideko Yamanaka

**Affiliations:** 1 Department of Analytical Chemistry, São Paulo State University (UNESP), 14800-900 Araraquara, SP, Brazil; hidekoy@iq.unesp.br; 2 Department of Natural Sciences, Federal University of Maranhão (UFMA), 65200-000 Pinheiro, MA, Brazil; 3 Department of Chemistry, Federal University of Maranhão (UFMA), 65080-805 São Luís, MA, Brazil; sakae.yn@gmail.com (S.Y.N.); rita.luz@ufma.br (R.d.C.S.L.); flavio.damos@ufma.br (F.S.D.); 4 UNESP, National Institute for Alternative Technologies of Detection, Toxicological Evaluation and Removal of Micropollutants and Radioactives (INCT-DATREM), UNESP, P.O. Box 355, 14800-900 Araraquara, SP, Brazil

**Keywords:** magnetic iron particles, organophosphorus pesticides, acetylcholinesterase, malathion

## Abstract

A renewable, disposable, low cost, and sensitive sensor for the detection of organophosphorus pesticides was constructed by immobilizing the acetylcholinesterase enzyme (AChE), via glutaraldehyde, on magnetic iron nanoparticles (Fe_3_O_4_) previously synthesized and functionalized with chitosan (CS). The sensor was denoted AChE/CS/Fe_3_O_4_. The magnetic nanoparticles were characterized by Fourier transform infrared spectroscopy and transmission electron microscopy. Acetylthiocholine (ATCh) was incubated with AChE/CS/Fe_3_O_4_ and attached to a screen-printed electrode using a magnet. The oxidation of thiocholine (from ATCh hydrolysis) was monitored at an applied potential of +0.5 V vs. Ag/AgCl(KCl_sat_) in 0.1 mol L^−1^ phosphate buffer solution (pH 7.5) as the supporting electrolyte. A mixture of the pesticide malathion and ATCh was investigated using the same procedure, and the results were compared and expressed as inhibition percentages. For determination of malathion, the proposed sensor presented a linear response in the range from 0.5 to 20 nmol L^−1^ (R = 0.9942). The limits of detection (LOD) and quantification (LOQ) were 0.3 and 0.8 nmol L^−1^, respectively. Real samples were also investigated, with recovery values of 96.0% and 108.3% obtained for tomato and pond water samples, respectively. The proposed sensor is a feasible option for malathion detection, offering a linear response, good sensitivity, and a low detection limit.

## 1. Introduction

Pesticides play an important role in increasing agricultural productivity [1,2]. Unfortunately, the excessive use of carbamates and organophosphates results in large amounts of residues of pesticides and their metabolites, which are harmful to the environment and human health [3]. The organophosphates (OPs) are among the most widely used classes of pesticides [4], and their toxic effects in vertebrates are well documented [5]. Their toxicity is due to inhibition of the activity of acetylcholinesterase (AChE), an enzyme that plays a role in controlling the transmission of nerve impulses to muscle and neuromuscular cells in living organisms [6]. 

Biosensors, which are analytical devices based on biological material immobilized on a transducer [7], offer certain advantages over other techniques such as chromatography, since they can provide quantitative information with a high level of accuracy, low cost, short response time, and minimal sample preparation. Acetylcholinesterase (AChE) is one of the biomolecules most widely investigated for the biosensor-based determination of pesticides [1,8].

The detection of pesticides by AChE involves the inhibition of AChE in the hydrolysis of acetylthiocholine (ATCh). The pesticide concentration can be accurately determined by monitoring the oxidation current of thiocholine (TCh) before and after the inhibition [2,9].

Biosensors based on inhibition of AChE are often used for the identification and quantification of pesticides. Although this method is not selective for different pesticides, it can provide information about the toxicological index, expressed as the total anticholinesterase load. Therefore, AChE-based biosensors are suitable as screening tools, providing a rapid response and signaling the presence of contaminants in samples, which are features that are vital for on-site measurements [10,11].

One of the most important steps in the development of an AChE enzyme-based sensor is the immobilization of the enzyme on the surface of the electrode while retaining the activity. The immobilization of AChE is governed by various interactions between the enzyme and the electrode material, which strongly affect the biosensor performance in terms of sensitivity, stability, response time, and reproducibility [12]. 

Several methods have been developed to immobilize enzymes, such as adsorption, crosslinking, covalence, and entrapment, among others, and the suitable selection of a supporting material can significantly improve the operational performance of the biosensor. The use of a chemically modified nanostructured electrode in an electrochemical biosensor can offer several advantages. Compared to bulk electrodes, the presence of nanoparticles on the surface of the electrode allows fast kinetics of electron transfer and increases the electroactive surface area, hence promoting redox reactions [13]. The increase in surface area allows lower limits of detection and greater sensitivity to analytes [14]. In particular, nanoparticles can increase the enzymatic load on the surface of the electrode [15], since the high surface-to-volume ratio increases the quantity of binding sites on the electrode surface, providing easier contact with the enzyme molecules. Chemically modified nanostructured electrodes optimize the use of the bioactive sites of the enzyme and provide pathways for electron transfer [12]. 

Although biosensors based on permanently modified surfaces are powerful devices for the detection of biomolecules, they are liable to fouling during continuous detection of analytes. 

Research concerning the synthesis and applications of magnetic iron nanoparticles has increased due to their potential technological applications, including in magnetic storage media, targeted drug delivery, contrast agents for magnetic resonance imaging, magnetic inks for jet printing, and biosensing. Magnetic iron nanoparticles have opened novel perspectives in bioelectrochemical analysis, because the recognition agent can be immobilized on the surfaces of the nanoparticles, with simple reconstruction of the sensor surface.

The present work describes the development of a methodology based on the interaction between AChE/CS/Fe_3_O_4_ and ATCh/malathion, followed by the attachment of the magnetic nanoparticles (mNPs) to the surface of a carbon screen-printed electrode (SPE), by means of a magnet, and evaluation of the inhibition of the enzyme. In the literature, usually, after the determination of pesticides, the electrodes are discarded, but in proposed methodology, the magnetic particles are washed away with water after taken the external magnet out, and then SPE is ready for another experiment. Although mNPs have been used for removal and quantification of rhodamine [16], as a glucose sensor [17], for the diagnosis of malaria [18], and for enzyme immunoassay atrazine sensor [19], concerning both the inhibition of AChE for pesticide detection and the use of iron magnetic particles for the AChE immobilization, only two articles were found in the literature [12,20].

## 2. Experimental

### 2.1. Reagents and Apparatus

All chemicals were analytical grade and were used without further purification. Acetylcholinesterase (AChE), acetylthiocholine chloride (ATCh), glutaraldehyde, chitosan (CS), malathion, iron(III) chloride hexahydrate, and iron(II) chloride tetrahydrate were purchased from Sigma-Aldrich. Glacial acetic acid was purchased from Isofar Indústria e Comércio Ltda (Rio of Janeiro, Brazil). Hydrochloric acid, sodium hydroxide, monobasic sodium phosphate, and disodium phosphate were obtained from Vetec Química Fina Ltda (Rio of Janeiro, Brazil). All solutions were prepared with water purified using a reverse osmosis system (OS10LXE, Gehaka Ltda, São Paulo, Brazil).

Fourier transform infrared spectroscopy (FTIR) analyses were performed using a Vertex 70 spectrometer equipped with a laser source and a DLaTGS detector (Bruker, Ettlingen, Germany), in the wavenumber range from 4000 to 400 cm^−1^. Transmission electron microscopy (TEM) analyses employed a CM200 superTWIN instrument (Philips/FEI, Eindhoven, The Netherlands). 

Electrochemical measurements were performed with an Autolab PGSTAT 128 N potentiostat/galvanostat (Metrohm Autolab B.V., Utrecht, The Netherlands) controlled by NOVA 2.0 software. Electrochemical detection was performed using screen-printed carbon electrodes (DropSens, Llanera, Asturias, Spain), with the magnetic nanoparticles coated with chitosan and the immobilized enzyme (AChE/CS/Fe_3_O_4_) being attached to the working surface of the electrode by means of an external magnetic field (after the interaction with the substrate or pesticide). After the measurement, the magnet was removed from the lower side of the SPE, all the AChE/CS/Fe_3_O_4_ was released, and the SPE was washed with water prior to use in another measurement. 

### 2.2. Synthesis of Nanoparticles

The synthesis of the Fe_3_O_4_ magnetic nanoparticles was carried out according to the methodology proposed by Ranjbari et al. [16]. Briefly, 5.2 g of FeCl_3_·6H_2_O, 2.0 g of FeCl_2_·4H_2_O, and 0.85 mL of HCl (12 M) were added to a round bottom flask and dissolved in 25 mL of deionized water. A 250 mL volume of 1.5 mol L^−1^ NaOH solution was heated at 80 °C and added dropwise to the solution containing Fe^2+^/Fe^3+^. The entire procedure was performed under a nitrogen atmosphere, with vigorous stirring in a water bath at 80 °C for 15 min. The precipitate was separated by applying a magnetic field, washed several times with deionized water, dried, and stored. 

### 2.3. Functionalization of the Nanoparticles with Chitosan (CS/Fe_3_O_4_)

The procedure for preparation of the chitosan-modified Fe_3_O_4_ nanoparticles (CS/Fe_3_O_4_) was performed following a methodology described in the literature [17]. Firstly, 0.1 g of chitosan was vigorously stirred in 10 mL of 1% (*v*/*v*) acetic acid solution for 50 min. Then, 0.5 g of Fe_3_O_4_ was added to the solution, with stirring for 50 min. The chitosan-modified Fe_3_O_4_ was recovered from the suspension, washed several times with deionized water, and stored in 10 mL of water at 4 °C.

### 2.4. AChE Functionalization of the Chitosan-Modified Fe_3_O_4_ Particles (AChE/CS/Fe_3_O_4_)

A 50 μL aliquot of the CS/Fe_3_O_4_ dispersion was transferred to a vial and the supernatant was discarded. Next, 20 μL of 25% glutaraldehyde solution, 20 μL of AChE solution (50 U/mL), 20 μL of ATCh solution, and 40 μL of phosphate buffer (PBS) were added to the CS/Fe_3_O_4_ dispersion, with stirring for 10 min, and the solid was recovered using a magnetic field. 

The electrochemical measurements were performed with a carbon-based screen-printed working electrode modified with 30 μL of AChE/CS/Fe_3_O_4_ particles (previously incubated with the substrate/pesticide), which were attached to the electrode using an external magnetic field.

### 2.5. Optimization of Experimental Conditions

The electrochemical response resulting from interaction between the AChE/CS/Fe_3_O_4_ particles and the enzymatic substrate (ATCh) was monitored using cyclic voltammetry (CV) and amperometry. Optimization was performed of the experimental parameters including substrate concentration, supporting electrolyte, pH, ionic strength of the medium, applied potential, and inhibition time. 

All measurements for the optimization of experimental conditions were performed in triplicate.

To determine the percentage enzyme inhibition (Inhibition (%)) of the proposed sensor, as a function of the time of incubation with the pesticide, 20 μL of 10 nmol L^−1^ malathion solution was dropped onto the surface of the screen-printed carbon electrode modified with AChE/CS/Fe_3_O_4_. Interaction between the AChE/CS/Fe_3_O_4_ and the pesticide was investigated using times from 2 to 12 min. The percentage inhibition was calculated using the following equation:

$$\mathrm{Inhibition}\;\left(\%\right)=\frac{I_{0}-I_{1}}{I_{0}}\;\times \;100$$

in which I_0_ is the peak current of the AChE/CS/Fe_3_O_4_-modified platform for thiocholine and I_1_ is the peak current for thiocholine after enzyme inhibition by malathion. After establishment of the incubation time, the analytical curve for the pesticide was constructed by varying the malathion concentration from 0.01 to 20 nmol L^−1^. The pesticide solutions were diluted in 0.1 mol L^−1^ phosphate buffer (pH 7.0). 

The AChE inhibition percentages were calculated by comparing the current intensities obtained for the same electrode, using the same medium and preconcentration time, in the presence and absence of the pesticide. All the measurements were carried out in triplicate. The limits of detection (LOD) and quantification (LOQ) of the method were calculated based on the parameters of the analytical curve, using LOD = 3*Sd/b* and LOQ = 10*Sd/b*, in which *Sd* is the standard deviation of the analytical curve and *b* is the angular coefficient [21]. 

### 2.6. Detection of Malathion in Tomato Sauce and Pond Water

A 1.5 g portion of tomato sauce purchased from a local market was mixed with 5 mL of distilled water, followed by centrifugation and collection of the supernatant for analysis. Pond water samples were collected on the campus of the Federal University of Maranhão (São Luís, MA, Brazil). The pH values of the tomato sauce and the pond water were adjusted to pH 7.0 with 0.1 mol L^−1^ NaOH solution. The samples were fortified with pesticide solution to a malathion concentration of (2.5 × 10^−7^, 7.5 × 10^−7^ and 1.2 × 10^−6^ mol L^−1^) and were then tested with the proposed sensor. 

## 3. Results and Discussion

### 3.1. Characterization of the Fe_3_O_4_ and CS/Fe_3_O_4_ Magnetic Nanoparticles

**Figure 1:**
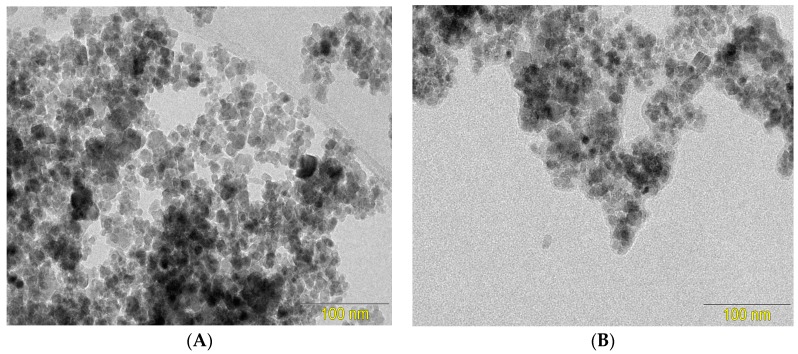
Transmission electron microscopy images of (**A**) Fe_3_O_4_ and (**B**) CS/Fe_3_O_4_.

The synthesized magnetic nanoparticles were characterized by Fourier transform infrared spectroscopy (FTIR) and transmission electron microscopy (TEM). Figure 1 shows TEM images of the magnetic nanoparticles, before (Figure 1A) and after modification with chitosan (Figure 1B). It can be seen that the magnetic nanoparticles formed aggregates, probably due to coercive forces and magnetic dipolar interaction among the particles. The micrographs revealed a change in the surface morphology of the Fe_3_O_4_ nanoparticles after the modification with chitosan, with formation of chitosan films around the particles. The mean Fe_3_O_4_/CS particle size was 13.2 nm. 

**Figure 2:**
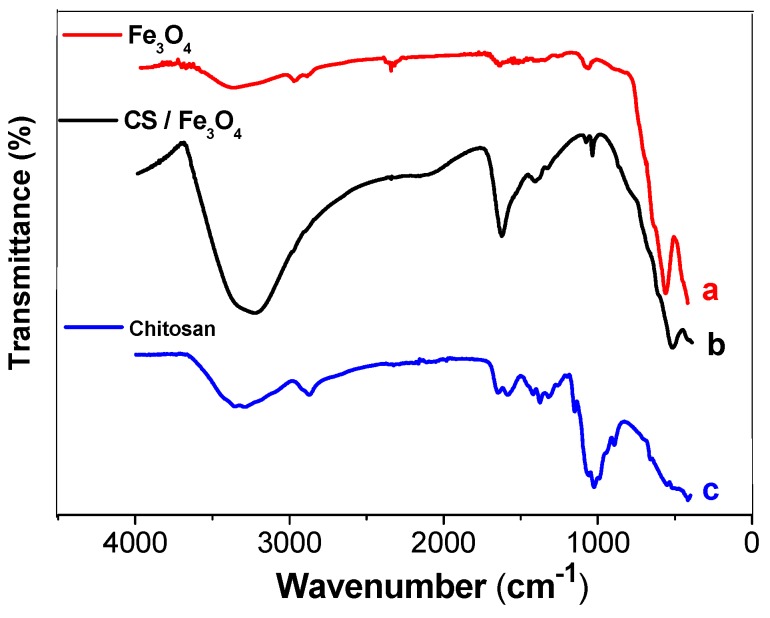
FTIR spectra of (a) Fe_3_O_4_, (b) chitosan, and (c) CS/Fe_3_O_4_.

Figure 2 shows FTIR spectra of the unmodified Fe_3_O_4_ NPs, chitosan, and the chitosan-functionalized Fe_3_O_4_ NPs. The spectrum for the unmodified Fe_3_O_4_ NPs (Figure 2a) showed a small and broad band at 3400 cm^−1^, corresponding to the O–H stretching bands of water, while a peak at around 541 cm^−1^ could be attributed to the characteristic stretching vibration of Fe–O bonds [22]. The spectrum of chitosan (Figure 2b) showed a large and broad absorption band at about 3400 cm^−1^, due to O–H stretching. In addition, the FTIR spectrum of chitosan presented a peak at about 1634 cm^−1^, corresponding to amino group (–NH_2_) bending vibration, and a peak at about 1041 cm^−1^, due to C–O–C stretching vibration. The spectrum of CS/Fe_3_O_4_ (Figure 2c) presented all the characteristic peaks and bands of the mNPs and chitosan. However, the peak corresponding to metal-oxygen binding in the Fe_3_O_4_/CS nanoparticles, at 541 cm^−1^, showed a small displacement to 526 cm^−1^, suggesting interaction between Fe_3_O_4_ and chitosan [17]. Therefore, the TEM and FTIR results indicated that the magnetic iron nanoparticles had been successfully coated with chitosan. 

### 3.2. Cyclic Voltammetry Evaluation of Thiocholine Oxidation on the Screen-Printed Carbon Electrode Modified with AChE/CS/Fe_3_O_4_

**Figure 3:**
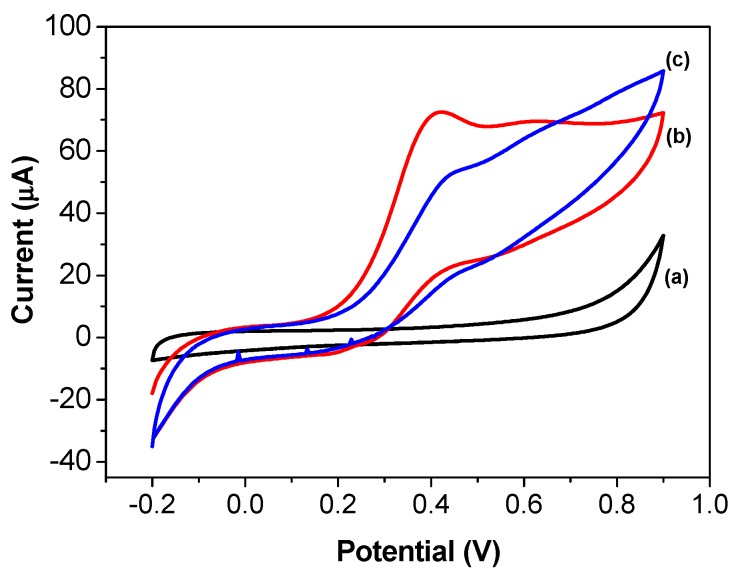
Cyclic voltammograms obtained using the screen-printed carbon electrode modified with AChE/CS/Fe_3_O_4_ in (a) 0.1 mol L^−1^ phosphate buffer solution at pH 7.4; (b) with addition of 20 mmol L^−1^ ATCh; and (c) with addition of 20 mmol L^−1^ ATCh plus 1.2 µmol L^−1^ of malathion.

Figure 3 shows the cyclic voltammograms obtained using the screen-printed carbon electrode modified with AChE/CS/Fe_3_O_4_. The electrode did not exhibit any faradaic electrochemical processes in 0.1 mol L^−1^ phosphate buffer solution at pH 7.4 (Figure 3a). However, it showed electrochemical activity when incubated in buffer solution containing 20 mmol L^−1^ of ATCh (Figure 3b). The peak at 0.41 V vs. Ag/AgCl corresponded to the oxidation of thiocholine, the product of ATCh hydrolysis catalyzed by AChE. After incubation of the AChE/CS/Fe_3_O_4_ with 1.2 µmol L^−1^ malathion, the peak current decreased, indicating an inhibitory effect of the pesticide on the activity of the AChE (Figure 3c). 

### 3.3. Optimization of Experimental Conditions

**Figure 4:**
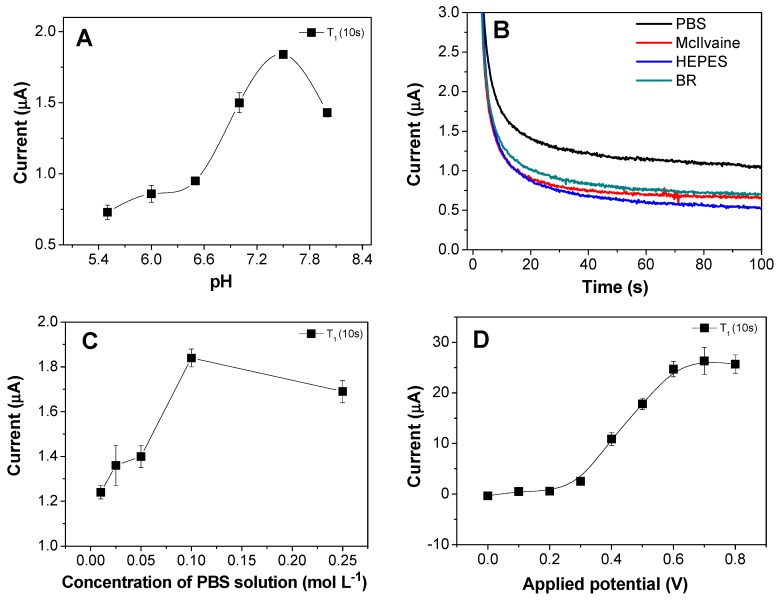
Effects of (**A**) pH, (**B**) nature of the buffer solution, (**C**) concentration of the buffer solution, and (**D**) applied potential on the response of the screen-printed carbon electrode modified with AChE/CS/Fe_3_O_4_.

The effects of the experimental parameters on the electrochemical response of the sensor were investigated. Some of the results are shown in Figure 4. 

The effect of the ATCh concentration (from 0.01 to 40 mmol L^−1^) on the electrochemical response of the screen-printed carbon electrode modified with AChE/CS/Fe_3_O_4_ was evaluated (data not shown). It was found that the peak current increased as the concentration of ATCh was increased up to 10 mmol L^−1^, followed by a decrease, indicating that the sensor had reached saturation. This was due to enzymatic saturation under conditions of high substrate concentration, with all the active sites of the enzyme becoming occupied by substrate molecules and/or products. The optimum ATCh concentration selected for use in the subsequent experiments was 10 mmol L^−1^.

The pH can affect the enzymatic activity in an aqueous solution [23]. Therefore, the influence of pH on the sensor response was investigated using a 0.1 mol L^−1^ phosphate buffer solution at pH from 5.8 to 9.0. The results (Figure 4A) showed that the highest peak current occurred at pH 7.5, which was close to the optimum pH for the activity of the non-immobilized AChE enzyme (pH 7.0) [24]. 

The oxidation of thiocholine was investigated in phosphate buffer solution (PBS), Mcllvaine buffer solution, HEPES buffer (*N*-2-hydroxyethyl) piperazine-*N*′-(2-ethanesulfonic acid), and Britton-Robinson buffer (BR). The results (Figure 4B) showed that the best electrochemical response was obtained in PBS, which could be attributed to the mobility of its ions. Investigation of the effect of the buffer concentration (Figure 4C) revealed that the highest sensitivity of the proposed sensor was achieved using 0.1 mol L^−1^ PBS. 

The potential applied to the working electrode also affected the sensitivity of the AChE/CS/Fe_3_O_4_-modified screen-printed carbon platform, since it could modulate the electrochemical energy levels of the electrode, hence improving the electrochemical response of the system. Figure 4D shows the effect of the applied potential in the range 0–0.8 V on the oxidation of thiocholine, with the maximum electrochemical response achieved at 0.5 V, after which the current remained constant. This potential was therefore sufficient to oxidize the thiocholine, while a higher applied potential could increase the contribution of other substances (interferents) to the total current.

The results showed that the optimal conditions for the immobilization of AChE, via glutaraldehyde, on iron nanoparticles that had been previously synthesized and functionalized with chitosan were as follows: 10 nmol L^−1^ of ATCh, 0.1 mol L^−1^ phosphate buffer solution at pH 7.5, and applied potential of 0.5 V. 

### 3.4. Temporal Behavior of Sensor Inhibition by Malathion

**Figure 5:**
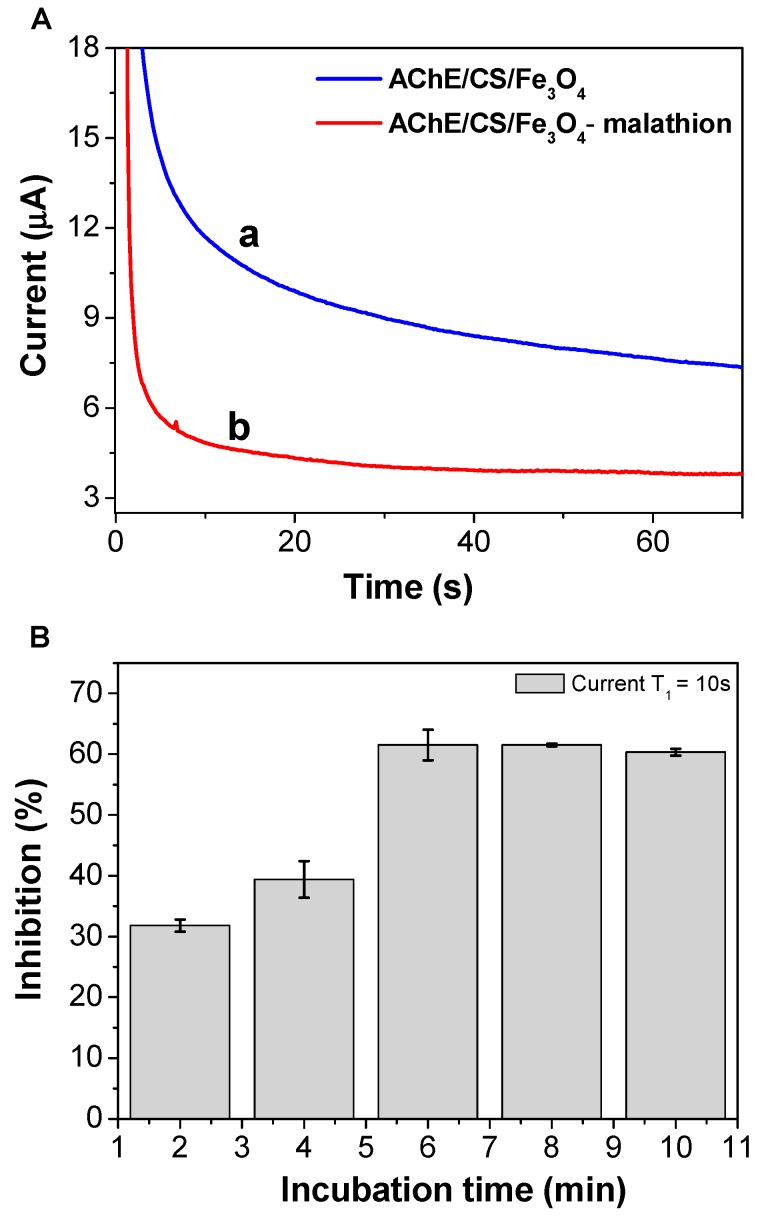
(**A**) Amperograms obtained using the screen-printed carbon electrode modified with AChE/CS/Fe_3_O_4_ magnetic nanoparticles (a) before and (b) after inhibition of AChE/CS/Fe_3_O_4_ in a solution containing 10 nmol L^−1^ of malathion. (**B**) Plot of inhibition (%) of the biosensor modified with AChE/CS/Fe_3_O_4_ according to incubation time.

Figure 5A shows the sensor response before and after inhibition of the AChE enzyme by malathion. The peak thiocholine oxidation current measured at 10 s decreased from an initial value of 11.7 μA to 4.8 μA after 12 min of exposure of the AChE/CS/Fe_3_O_4_ particles to the pesticide. The decrease in the peak current could be explained by the fact that malathion belongs to the OP class of pesticides, which react with AChE to form complexes. The interaction between OPs and AChE-modified magnetic nanoparticles results in an irreversible inhibition of AChE activity and decreased enzymatic capacity to produce thiocholine [25]. As shown in Figure 5B, there was an increasing in the percentage inhibition of the AChE/CS/Fe_3_O_4_ particles up to 6 min, after which the electrochemical response did not change significantly. Considering the 61.6% inhibition obtained for the sensor after incubation in 10 nmol L^−1^ malathion solution, a time of 6 min was selected in the subsequent experiments.

### 3.5. Analytical Performance of the AChE/CS/Fe_3_O_4_-Modified Screen-Printed Carbon Electrode for Determination of Malathion Pesticide

**Figure 6:**
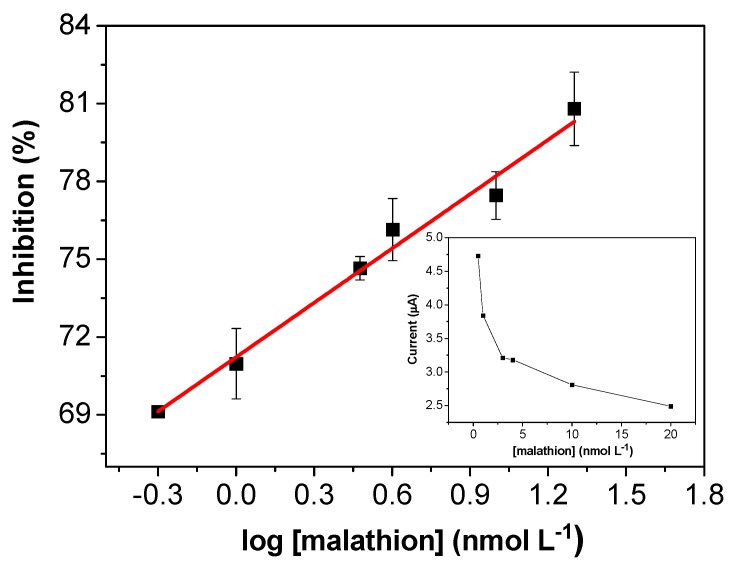
Calibration plot for the malathion biosensor in the range from 0.5 to 20 nmol L^−1^. Insert: plot of current according to malathion concentration.

After optimization of the experimental parameters, malathion was used as an analyte to evaluate the response of the modified electrode. The sensitivity of the sensor was investigated by chronoamperometry, with determination of the current signal in the presence of the pesticide at different concentrations. The analytical curve for the determination of malathion (Figure 6) presented a linear range from 0.5 to 20 nmol L^−1^ and could be described by the following equation: I (%) = [71.2 (±0.2) + 7.0 (±0.4)]/log [malathion] (nmol L^−1^), with R = 0.9942 (*n* = 6). The calculated limits of detection and quantification were 0.3 and 0.8 nmol L^−1^, respectively. The relation between the current and the concentration is shown in the inset of Figure 6. As can be seen, the current gradually decreased as the malathion concentration increased, due to the inhibitory effect of the pesticide on the enzymatic activity. 

Previous studies of the development of biosensors using AChE-modified screen-printed electrodes with inhibition by pesticides have reported LODs ranging from 7.3 to 5 × 10^3^ nmol L^−1^, depending on the type of immobilization and the analyte studied [26,27,28,29,30,31]. The literature also reports the modification of printed electrodes for immobilization of AChE using Prussian blue [10,32] multi-walled carbon nanotubes [33], and electropolymerization [34], with LODs in the range from 19 to 126 nmol L^−1^. These values were much higher than the LOD of 0.3 nmol L^−1^ obtained in the present work, showing that the method for AChE enzyme immobilization on chitosan-functionalized nanoparticles was highly promising.

The performance of the proposed sensor was compared with other sensors (Table 1) in which AChE was immobilized on nanoparticles. Only the works of Jeyapragasam et al. [12] and Kostelnik et al. [20] involved the use of magnetic nanoparticles for pesticide determination. Although the pesticides listed in Table 1 are different, they all belong to the organophosphate or carbamate classes. The results indicated that the proposed sensor offered a reasonable linear range for pesticide detection and that the detection limit was lower than for some of the methods reported in the literature except on [35,36]. In reference 35, the limit of detection was calculated considering 2–3% inhibition instead of 3*Sd/b* of analytical curve as on the present manuscript, and in reference 36 the high sensitivity is caused by the genetically-modified AChE and amplification of current by the Fe-Ni nanoparticles.

### 3.6. Determination of Malathion in Tomato Sauce and Pond Water Samples

The AChE/CS/Fe_3_O_4_-modified screen-printed carbon electrode was used for the detection of malathion in samples of tomato sauce and pond water, achieving recoveries of 96.0 to 108.3% (Table 2). The results indicated that the proposed sensor could be satisfactorily used for such analyses.

## 4. Conclusions

A simple and effective strategy was used to immobilize AChE on magnetic iron nanoparticles, enabling the development of a sensitive sensor for the detection of malathion pesticide. 

Magnetic Fe_3_O_4_ nanoparticles coated with the natural polymer chitosan were successfully prepared and used for the immobilization of AChE, employing glutaraldehyde as a crosslinking reagent. The immobilized AChE was put in contact with the pesticide and then was fixed onto SPE through external magnet, and the current was measured. Such a strategy was important to develop a sensitive methodology, another advantage, as the magnetic particles were washed away with water after taking the external magnet out, and then the SPE was ready for further experimentation. On the construction of the analytical curve, the repeatability between the responses of sensors was 1.0%.

The proposed sensor provides a lower limit of detection, compared to other published methods involving the immobilization of AChE, and is suitable for the determination of malathion.

## Figures and Tables

**Table 1:** Comparison of the analytical performance of sensors for the detection of pesticide residues.

AChE Based Biosensor	Pesticide	Technique	Linear Response Range (nmol L^−1^)	LOD (nmol L^−1^)	Ref.
AChE/Fe_3_O_4_–CH/GCE	Carbofuran	SWV/CV	5.0–90	3.6	[12]
MPs-AChE	Carbofuran	SWV	39–625	20	[20]
AChE-AuNPs-CaCO_3_	Malathion/C	EIS/Amperometry	0.1–100	0.1	[35]
PVA-AWP/Fe-Ni/NP AChE	Phosmet-oxon	CV/Amperometry	0.1–5	0.1	[36]
AChE–AuNPs/MPS/Au	Carbamate	CV/Amperometry	3–2 × 10^3^	1.0	[37]
Nano-silver/AChE/chitosan	Paraoxon	CV/Amperometry	0–3.63 × 10^2^	14.5	[38]
AChE/CS/Fe_3_O_4_	Malathion	CV/Amperometry	0.5–20	0.3	This work

CH—chitosan hydrogen chloride; MPS—(3-mercaptopropyl)-trimethoxysilane; AuNPs—gold nanoparticles; PVA-AWP—azide-unit water-pendant polyvinyl alcohol; Fe-Ni—iron-nickel; NP—nanopowder; MPs—magnetic particles (Fe_3_O_4_); C—Chlorpyrifos; SWV—square wave voltammetry; EIS—electrochemical impedance spectroscopy; GCE—glassy carbon electrode.

**Table 2:** Recoveries of malathion from tomato sauce and pond water samples.

Samples	Malathion Added (mol L^−1^)	Malathion Expected (mol L^−1^)	Malathion Found (mol L^−1^)	Recovery (%)
Tomato sauce	0	0	-	-
	2.5 × 10^−7^	2.5 × 10^−7^	2.7 × 10^−7^	108.0 (±1.95) ^a^
	7.5 × 10^−7^	7.5 × 10^−7^	7.4 × 10^−7^	98.7 (±2.65) ^a^
	1.2 × 10^−6^	1.2 × 10^−6^	1.2 × 10^−6^	100.0 (±2.66) ^a^
Pond water	0	0	-	-
	2.5 × 10^−7^	2.5 × 10^−7^	2.4 × 10^−7^	96.0 (±3.40) ^a^
	7.5 × 10^−7^	7.5 × 10^−7^	7.5 × 10^−7^	100.0 (±1.01) ^a^
	1.2 × 10^−6^	1.2 × 10^−6^	1.3 × 10^−6^	108.3 (±0.87) ^a^

^a^ Standard deviation of three replicates.
